# Thermally Bistable Stiff Stilbene Photoswitches and Polymer Applications

**DOI:** 10.1002/tcr.70143

**Published:** 2026-04-14

**Authors:** Keiichi Imato

**Affiliations:** ^1^ Applied Chemistry Program Graduate School of Advanced Science and Engineering Hiroshima University Higashihiroshima Japan

**Keywords:** isomerization, mechanophores, molecular machines, photoswitches, polymers

## Abstract

Photoresponsive molecular switches (photoswitches) enable reversible control of molecular structures and properties using light, offering powerful opportunities for functional materials. However, their practical use in polymeric systems has long been constrained by thermal instability, which also hampers mechanistic understanding and precise control. This Personal Account summarizes recent progress in thermally stable photoswitches, with particular emphasis on stiff stilbene (SS) and sterically hindered stiff stilbene (HSS) photoswitches, largely based on our own studies. After outlining the fundamental characteristics of rare thermally stable photoswitches, it is shown how HSS photoswitches combine large structural changes with exceptional thermal bistability and well‐balanced photoisomerization yields. These unique features have led to diverse polymer applications, including mechanochemical studies, reversible control of single‐chain conformations, and consequent switching of inter‐ and intrachain interactions, solubility, and surface wettability. Finally, future prospects for thermally stable photoswitches in polymer science are briefly discussed.

## Graphical abstract

Thermally stable photoswitches offer new opportunities for controlling polymer structures and functions under nonequilibrium conditions. This Personal Account highlights stiff stilbene (SS) and sterically hindered stiff stilbene (HSS) photoswitches, which combine large structural changes with exceptional thermal bistability. Their unique properties have enabled diverse polymer applications and unusual photoresponsive behavior.

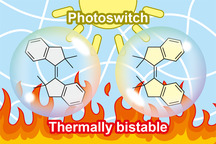

## 1 Introduction

**FIGURE 1:**
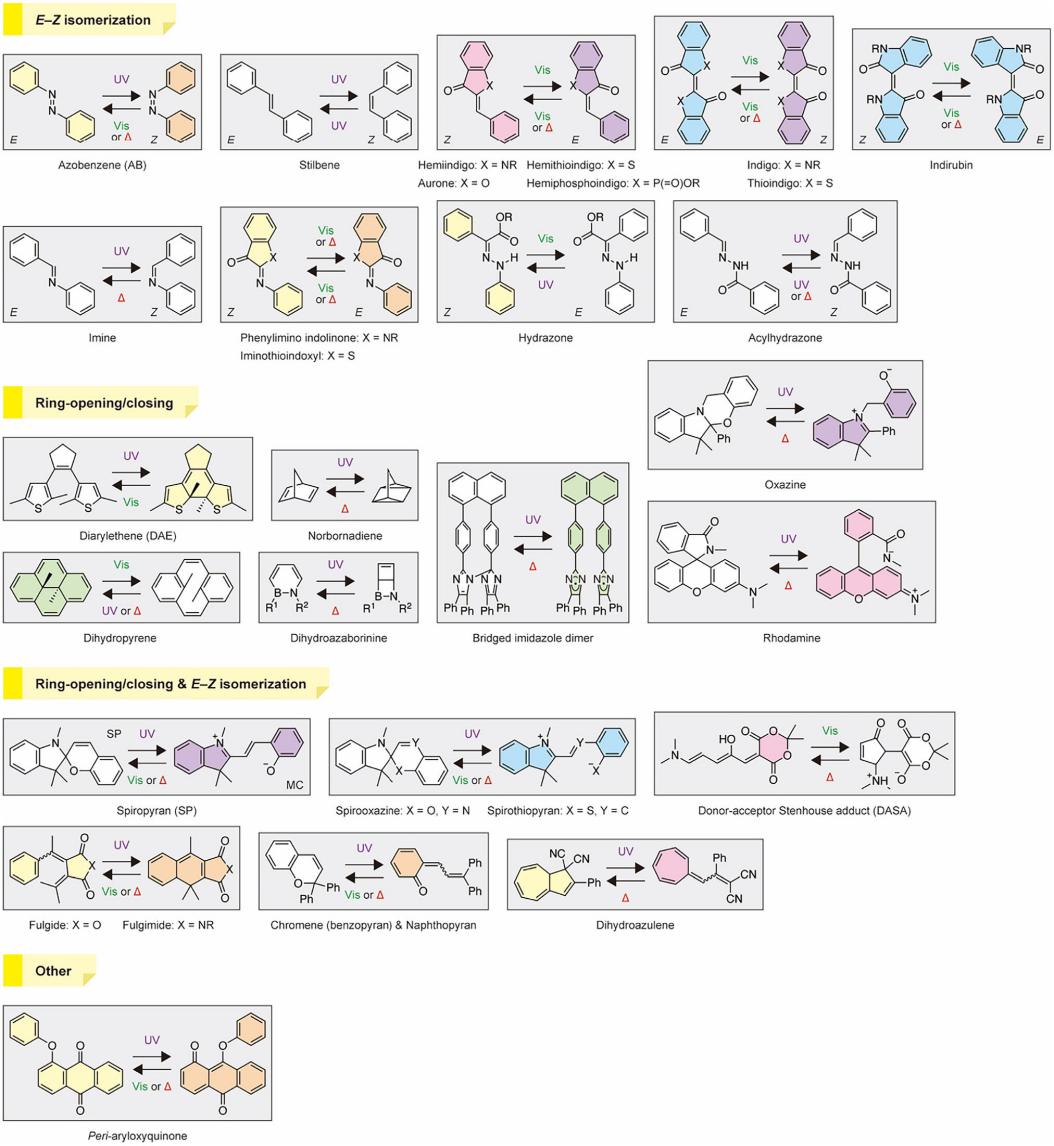
Chemical structures (drawn using typical colors in solution) and isomerization of representative photoswitches.

**FIGURE 2:**
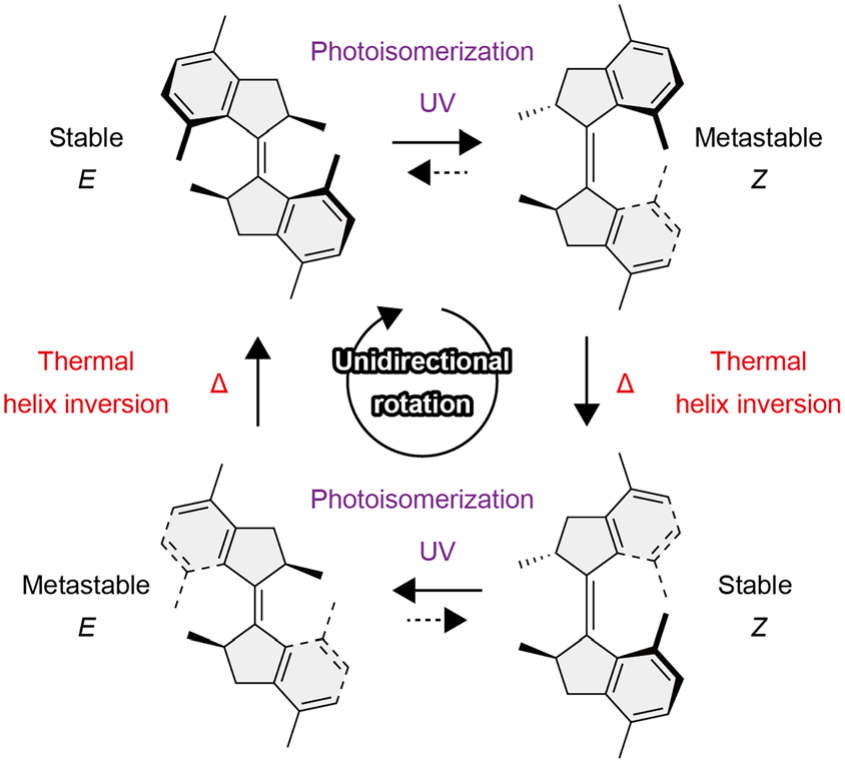
Unidirectional rotation of overcrowded alkene‐based first‐generation molecular motor.

Photoresponsive molecular systems have attracted significant interest across a wide range of fields because light offers distinct advantages: it can rapidly and selectively stimulate only target materials, regions, or molecules in a non‐contact manner, with high spatiotemporal precision and facile, precise control over wavelength and intensity. In particular, photoresponsive molecular switches (photoswitches), which interconvert between at least two thermodynamically (meta)stable isomers upon wavelength‐selective photoirradiation, are intriguing because they enable reversible changes in molecular structures and physicochemical properties. In general, molecules capable of undergoing photoinduced isomerization in at least one direction are classified as photoswitches. A wide variety of photoswitches exhibiting distinct structural and physicochemical changes have been reported [1, 2, 3, 4, 5, 6]. Representative examples are shown in Figure 1. Among these, azobenzenes (ABs) represent the most widely used family of photoswitches and are characterized by large structural changes upon *E*–*Z* isomerization [7, 8, 9, 10, 11, 12, 13, 14]. Diarylethenes (DAEs) undergo drastic changes in conjugation upon photoisomerization between their open‐ring and closed‐ring forms [15, 16, 17, 18, 19]. Spiropyrans (SPs), which are nonpolar, neutral, colorless, and nonfluorescent, photoreversibly transform into highly polar, zwitterionic, colored, and weakly fluorescent merocyanines (MCs) through *E*–*Z* isomerization about the C=C double bonds and ring‐opening/ring‐closing reactions [20, 21, 22, 23, 24, 25]. DAEs and SPs represent the next most commonly employed photoswitches after ABs. Stilbenes undergo reversible *E*–*Z* photoisomerization, as do ABs; however, the *Z* isomer can cyclize either reversibly, as in DAEs, or irreversibly in the presence of oxidants such as oxygen [26, 27]. (Thio)indigos [28, 29, 30, 31, 32, 33], indirubin [34], hemi(thio/phospho)indigos [30, 35, 36, 37, 38, 39, 40, 41, 42, 43, 44, 45, 46, 47, 48], aurones [49], (*α*‐bis)imines [50, 51, 52, 53, 54], phenylimino indolinones [55], iminothioindoxyls [56, 57, 58], and (acyl)hydrazones [59, 60, 61, 62, 63, 64, 65, 66, 67] interconvert between *E* and *Z* isomers, as do ABs. Dihydropyrenes [68, 69, 70, 71], dihydroazaborinines [72, 73], and norbornadienes [74, 75, 76] exhibit photoinduced pericyclic ring‐opening and ring‐closing reactions, as do DAEs. The ring‐opening and ring‐closing processes of bridged imidazole dimers involve homolytic bond cleavage [77, 78, 79, 80, 81, 82, 83, 84], whereas those of oxazines [85, 86, 87] and rhodamines [88, 89] proceed via heterolytic bond cleavage. Spirooxazines [22, 23], spirothiopyrans [23, 90, 91], fulgides [92, 93, 94, 95], fulgimides [94, 95], chromenes (benzopyrans)/naphthopyrans [96, 97, 98, 99, 100, 101], dihydroazulenes [102, 103, 104], and donor–acceptor Stenhouse adducts (DASAs) [105, 106, 107, 108, 109, 110, 111, 112, 113, 114] undergo interconversion between open‐ring and closed‐ring forms, accompanied by *E*–*Z* isomerization, as do SPs. In contrast to these mechanisms, *peri*‐aryloxyquinones isomerize through aryl migration upon light irradiation [115]. Beyond these examples, photoinduced conformationally transformable molecules, such as flapping molecules (FLAPs) [116, 117], 1,2‐diketones [118, 119, 120], tetra‐BF_2_ complexes [121], and phenothiazine derivatives [122], can serve as photoswitches. Light‐driven molecular motors [123, 124, 125, 126, 127] based on overcrowded alkenes [128, 129, 130, 131, 132, 133], imines [134, 135], hemithioindigos [136, 137, 138, 139], oxindoles [140], retinal [141], pyrrolidinones [142], and barbituric acids [143] can also be regarded as photoswitches; however, as molecular machines, they exhibit more complex functional behavior and should be considered part of a higher‐level category (Figure 2). A growing number of distinct new photoswitches have continued to emerge in recent years [144, 145, 146, 147, 148, 149, 150, 151, 152, 153, 154, 155].

**FIGURE 3:**
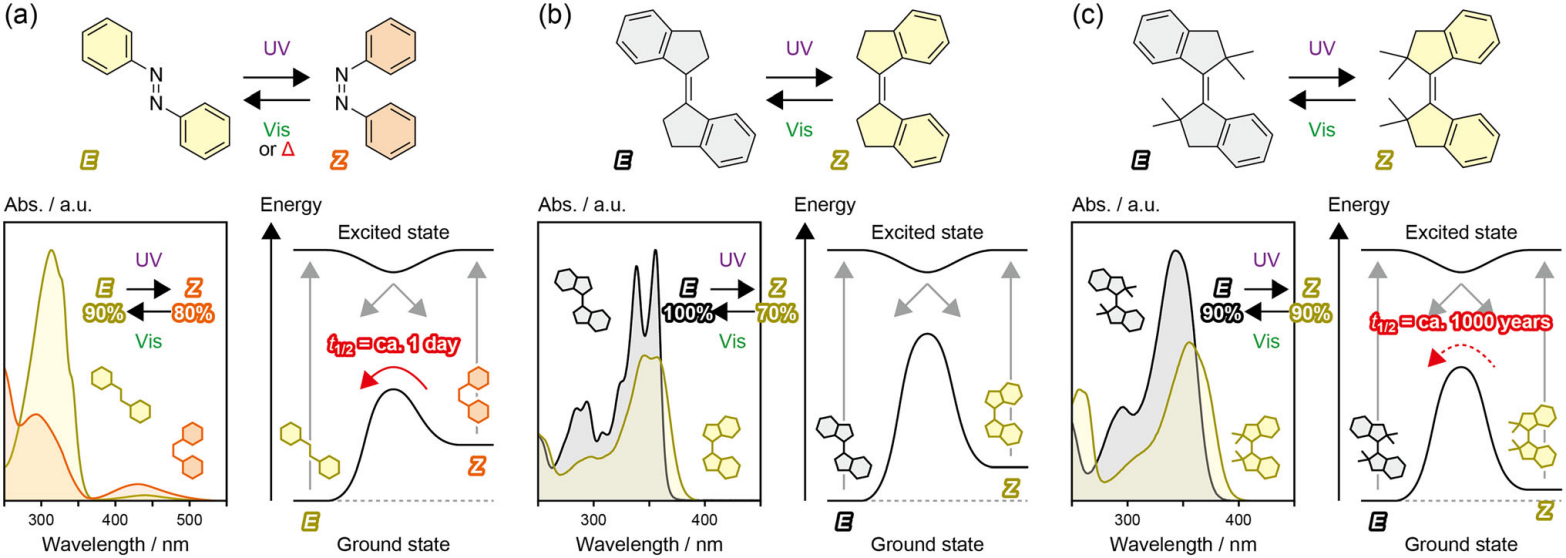
Chemical structures, representative photoabsorption spectra, and energy diagrams for *E*–*Z* isomerization of (a) AB, (b) SS, and (c) HSS photoswitches.

The photoswitches described above have enabled a broad array of fascinating and diverse applications, including other molecular machines, actuators, photoinduced transitions between solid/glass and liquid states or liquid‐crystalline phases, photoswitchable adhesives, solar energy storage and release, supramolecular systems, cages, frameworks, biological systems, photopharmacology, biomaterials, catalysts, additive manufacturing, and optoelectronic devices [156, 157, 158, 159, 160, 161, 162, 163, 164, 165, 166, 167, 168, 169, 170, 171, 172, 173, 174, 175, 176, 177, 178, 179, 180, 181, 182, 183, 184, 185, 186, 187]. Among these, polymer applications have been particularly intensively investigated because of their significance in both academic and industrial contexts [188, 189, 190]. A central limitation, however, is thermal instability: at room temperature (RT), one isomer spontaneously converts to another, which can be detrimental for certain uses. For example, the metastable *Z* isomer of the parent AB thermally isomerizes to the *E* isomer rapidly even at RT, with a half‐life (*t*_1/2_) of less than 1  day (Figure 3a) [7, 8, 9, 10, 11, 12, 13, 14]. This half‐life can be prolonged to several decades through skeletal modifications [9, 10, 11, 12, 13, 14], such as the introduction of *ortho*‐substituents [191, 192, 193, 194, 195, 196] and heteroaryl groups [197, 198, 199, 200, 201, 202, 203, 204, 205]. Meanwhile, in some instances, these modifications shorten the half‐life but instead extend absorption into the visible and near‐infrared regions [9, 10, 11, 12, 13, 14], as observed for ring‐bridged [206, 207, 208, 209, 210, 211], BF_2_‐complexed [212, 213, 214], and ionic [215, 216, 217, 218] derivatives. Such instability hampers deep understanding of photoswitching behavior in polymeric materials, precise control over this behavior, elucidation of its effects on polymer chains and materials, and the scope and practicality of applications. Thermally stable photoswitches remain scarce. This account reviews a handful of thermally stable photoswitches and their polymer applications, with a primary focus on our own studies.

## 2 Thermally Bistable Stiff Stilbene Photoswitches

Among the wide variety of photoswitches, truly thermally stable systems—e.g., those whose metastable isomer has a *t*_1/2_ exceeding 100  years at RT—have been identified only in DAEs [15, 16, 17, 18, 19], hemi(thio/phospho)indigos [30, 35, 36, 37, 38, 39, 40, 41, 42, 43, 44, 45, 46, 47, 48], and hydrazones [61, 62, 63, 64, 65, 66, 67] (Figure 1). These thermally bistable photoswitches have enabled deeper investigation of their fundamental chemistry in materials and have inspired new applications.

During my doctoral work and the early stage of my academic career as a postdoctoral researcher and assistant professor, I primarily investigated mechanophores—mechanically active molecules—and functional polymeric materials based on dynamic covalent and supramolecular chemistries [219, 220, 221, 222, 223, 224, 225, 226, 227, 228, 229, 230, 231, 232, 233, 234, 235]. After receiving my Ph.D., I began exploring photoswitches such as ABs, DAEs, and SPs, with a particular interest in biomaterial applications [236, 237, 238, 239, 240, 241]. However, detailed investigation of AB and SP photoswitches in materials was hampered by their thermal instability, which limited mechanistic insight into photoswitch–material interactions. I recognized the limitations of thermally unstable photoswitches for uncovering the fundamental chemistry within materials and for pursuing practical uses. Moreover, the comparatively small structural changes accompanying isomerization of thermally bistable DAE photoswitches seemed unsuitable for certain polymeric systems. Motivated by these challenges, since joining Hiroshima University in 2019, we have focused on thermally bistable photoswitches that undergo large structural changes akin to those of ABs. This direction aims to bridge the gap between molecular behavior and material function, providing deeper molecular‐level understanding of photoinduced transformations in polymeric systems.

As noted above, thermally stable photoswitch scaffolds were quite limited, and only a handful of papers had appeared when we began this line of research. Through extensive literature surveys, we became fascinated by stiff stilbene (SS) photoswitches, five‐membered ring‐fused analogs of stilbenes that exhibit both large geometrical changes and remarkable thermal bistability (Figure 3b) [242]. Four‐, six‐, and seven‐membered ring‐fused stilbenes may be regarded as SSs but have infrequently been used, likely because of low photoisomerization yields (substantial overlap of the *E* and *Z* photoabsorption bands) and facile photoinduced electrocyclization [243, 244]. Here, “SS” refers specifically to five‐membered ring‐fused stilbene. The parent SS can be synthesized in a single step from a commercially available reagent via the established McMurry coupling [242], and various substituents have been introduced onto the skeleton [245, 246, 247]. The *Z* isomer exists as two helically twisted enantiomers with right‐handed and left‐handed helicity (*P* and *M*, respectively), and the energy barrier (Δ*G* ^‡^) between them is low enough to allow rapid interconversion at RT [248]. The *E*‐to‐*Z* and *Z*‐to‐*E* isomerizations proceed upon irradiation with UV light and visible light, respectively. In SSs (five‐membered ring‐fused stilbenes), cyclization is structurally prohibited by their rigid framework, although oxidative degradation via other pathways has been observed [249, 250]. The *Z* isomer is considered metastable; however, to the best of our knowledge, the free energy difference between the isomers (Δ*G*) and Δ*G* ^‡^ for thermal *Z*‐to‐*E* isomerization have not yet been experimentally determined [251]. When an SS derivative was heated above 140°C in dimethyl sulfoxide (DMSO), decomposition and solvent evaporation occurred before thermal isomerization could be observed [252]. The *E*–*Z* isomerization can also be effectively catalyzed by an electric field [253]. Earlier studies mainly addressed the photophysics of SSs, in parallel with work on stilbenes [254, 255, 256, 257]. More recently, their intriguing applications have been explored in molecular force probes [258, 259, 260, 261, 262], catalysis [263, 264, 265, 266], and supramolecular [267, 268, 269, 270, 271, 272, 273, 274], biomolecular [275, 276, 277, 278, 279, 280], and molecular‐machinery [281, 282, 283, 284, 285] systems. However, the central C=C double bond of SSs is highly reactive toward Grubbs catalysts and toward radicals—leading to *E*–*Z* isomerization and loss of photoswitching capability, respectively—which complicates polymer incorporation [286, 287]. To date, only five reports on polymers containing SS photoswitches have appeared [286, 288, 289, 290, 291]. Furthermore, the relatively low *E*‐to‐*Z* photoisomerization yield (<70%) is a drawback, although the *Z*‐to‐*E* photoisomerization proceeds nearly quantitatively [242]. These limitations ultimately led us to sterically hindered stiff stilbene (HSS) photoswitches (Figure 3c), which offered a way forward.

HSS is an SS derivative substituted with four methyl groups at the C2 and C2′ positions. Mono‐alkylated variants at each C2 and C2′ position are known as first‐generation molecular motors (Figure 2) [123, 124, 125, 126, 127, 128, 130, 132, 133]. Steric hindrance around the central C=C double bond confers tolerance toward Grubbs catalysts and toward radicals [287, 292]. Larger substituents—for example, four ethyl groups—could scarcely be installed at these positions in the synthesis via McMurry coupling (unpublished data). To the best of our knowledge, HSS derivatives with ring sizes other than five‐ and six‐membered rings have not been described. As with SSs, six‐membered ring‐fused HSSs have rarely been employed, likely because their *Z* isomers are unstable and spontaneously isomerize to the *E* isomers at RT owing to a significantly low energy barrier [293]. Five‐membered ring‐fused HSS photoswitches were first introduced by Prof. Shinmyozu's group in 2007 [294, 295], but no subsequent studies on HSSs appeared prior to our publication in 2022 [252]. The pioneering papers noted a large separation between the *E* and *Z* photoabsorption bands and stated that “no isomerization occurred during benzene reflux for the *Z* isomers.” Nevertheless, a comprehensive evaluation of HSS as a photoswitch had not been undertaken. We therefore decided to investigate HSS photoswitches in detail. We synthesized (five‐membered ring‐fused) SS and HSS derivatives and compared their performance as photoswitches with that of the corresponding AB derivative as a benchmark, because ABs are the most widely used photoswitches and, like SS and HSS photoswitches, are characterized by large structural changes (Figure 3) [252]. Density functional theory (DFT) calculations and reported single‐crystal structures indicated that HSS undergoes larger structural changes than AB and SS upon isomerization. The HSS photoswitch exhibited high and well‐balanced photoisomerization yields of approximately 90% in both *E*–*Z* directions as well as good photofatigue resistance upon irradiation with 300 nm UV light and 405 nm visible light. Furthermore, van’t Hoff and Eyring analyses of the thermal *Z*‐to‐*E* isomerization above 120°C extrapolated small Δ*G*_*Z*‐to‐*E*_ (−2.33 kJ  mol^−1^ at 25°C) and large Δ*G*_*Z*‐to‐*E*_^‡^ (131 kJ  mol^−1^ at 25°C) values, demonstrating the high thermal stability of the metastable *Z* isomer with a *t*_1/2_ of approximately 1000  years at RT. Apart from our work, substituent effects on photoswitching performance and biomolecular applications of HSSs have only very recently been examined [246, 296, 297].

## 3 Polymer Applications

As with thermally unstable photoswitches such as ABs and SPs, thermally stable photoswitches have also been physically dispersed in polymer matrices and chemically incorporated into polymer structures. A wide variety of polymer applications of DAE photoswitches has been explored over the past several decades [17, 18, 19]. For an overview of these developments, readers are referred to a recent comprehensive review [298]. On the other hand, thermally stable hemiindigo [45, 299], hydrazone [300, 301, 302, 303, 304, 305, 306, 307], and SS [286, 288, 289, 290, 291] photoswitches have only recently been employed in polymer applications. Hemiindigo photoswitches have been blended with common polymer matrices to afford photochromic polymeric materials capable of reversible optical inscription and erasure [45, 299]. Similarly, hydrazone photoswitches have been dispersed in poly(methyl methacrylate) and polybutadiene, demonstrating reversible optical printing of transparent but invisible multicolor fluorescent patterns [302] and complex fluorescent images [305]. They have also been chemically incorporated into the main chains of linear polyenes and the side chains of linear poly(meth)acrylates to transform their macromolecular conformations and to modulate their glass transition temperatures (*T*_g_s) [301, 306]. Liquid‐crystalline polymer networks containing hydrazone photoswitches in the side chains or at the cross‐linking points have enabled light‐driven actuators [300], optical information encryption [303], and photonic coatings [307]. Hydrazone photoswitches covalently attached to covalent organic frameworks have also been investigated [304]. SS photoswitches have been embedded into the side chains of linear polymers to develop a light‐controlled artificial K^+^ transport channel in liposomes and cancer cells [289] and into the main chain of a linear polymer to demonstrate light‐controlled living crystallization‐driven self‐assembly for nanoplatelets [291]. A single SS derivative has also been installed as a mechanophore—a molecule responsive to mechanical force—into the backbones of linear polystyrenes to investigate fragmentation of macromolecular solutes in rapid flows [288]. Ring‐opening metathesis polymerization of an SS macrocycle has been achieved by utilizing the ring strain generated in the macrocycle through *Z*‐to‐*E* photoisomerization [286]. Such light‐triggered modulation of the ring strain in other SS macrocycles has enabled switching between ring‐opening polymerization and ring‐closing depolymerization [290]. As with these thermally stable photoswitches, polymer applications of HSS photoswitches have so far been limited to our recent studies [287, 292].

**FIGURE 4:**
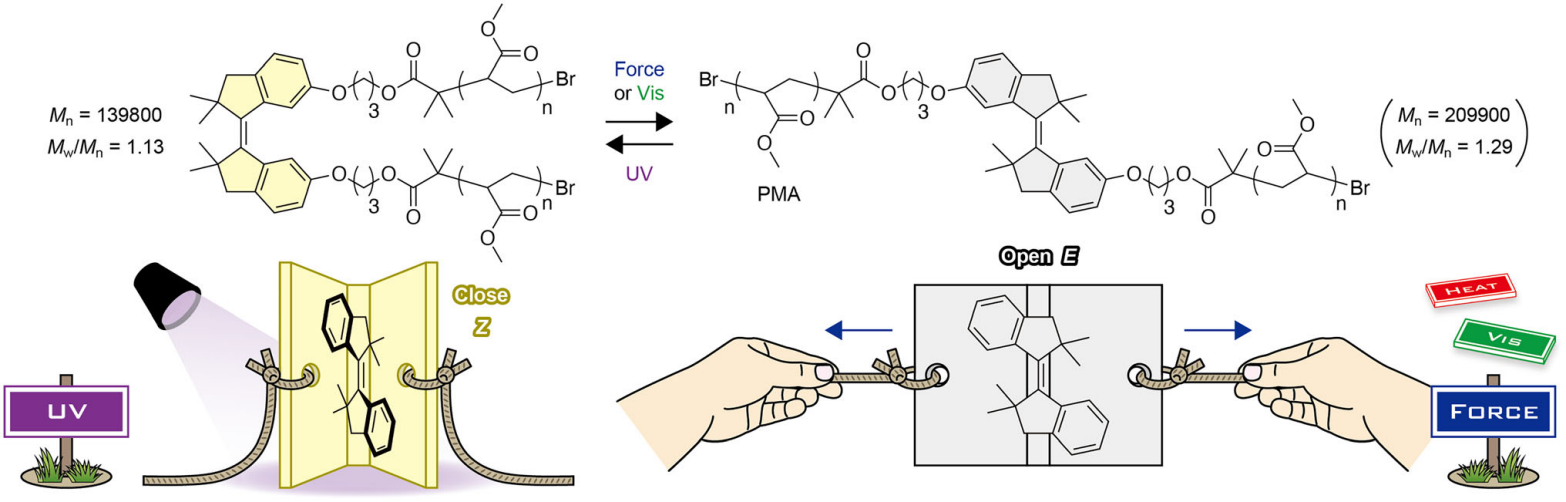
Chemical structures of linear polymers with a single *E* or *Z* isomer of HSS incorporated at the chain center, and their photochemical and mechanochemical isomerization.

First, we incorporated an HSS photoswitch at the center of polymer chains to transmit mechanical force through the polymer backbone and to investigate its mechanochemical reactivity (Figure 4) [287]. Some photoswitches are known to act as mechanophores—i.e., to undergo mechanically induced, direction‐selective isomerization—and mechanochemical reactivity determines their potential applications [234]. Owing to the steric hindrance of HSS, radical polymerization could be used for its incorporation without deteriorating the photoswitching capability, which was otherwise lost in incorporation of an SS photoswitch because the exposed central C=C double bond readily reacted with radicals. Atom transfer radical polymerization (ATRP), a living radical polymerization technique, was employed to obtain linear poly(methyl acrylate)s (PMAs) containing either the *E* or *Z* isomer of HSS at the chain center, with a number‐average molecular weight (*M*_n_) exceeding 100,000 and a dispersity (*M*_w_/*M*_n_) below 1.3. We used ultrasonication of polymer solutions to generate elongational force near the chain center. Ultrasonic irradiation of a 100% *E*‐PMA solution did not induce *E*‐to‐*Z* isomerization, whereas irradiation of a 100% *Z*‐PMA solution triggered *Z*‐to‐*E* isomerization. In addition, an 80% *E*‐PMA solution, obtained at the photostationary state (PSS) under 405 nm visible light irradiation, also underwent *Z*‐to‐*E* isomerization upon ultrasonication. By contrast, no *Z*‐to‐*E* isomerization was observed in a control mixed solution of a pure PMA without HSS and a low‐molecular‐weight HSS derivative, thereby excluding the possibility of thermal isomerization. Taken together, these observations suggest that HSS is a mechanophore that undergoes mechanochemical isomerization exclusively in the *Z*‐to‐*E* direction under elongational force. Because HSS behaves like a molecular hinge and exhibits reversible mechanochemical isomerization in combination with 340 nm UV light irradiation, it can be regarded as a photomechanically driven molecular hinge.

Around the same time as our report, SS was also demonstrated to function as a mechanophore [288].

**FIGURE 5:**
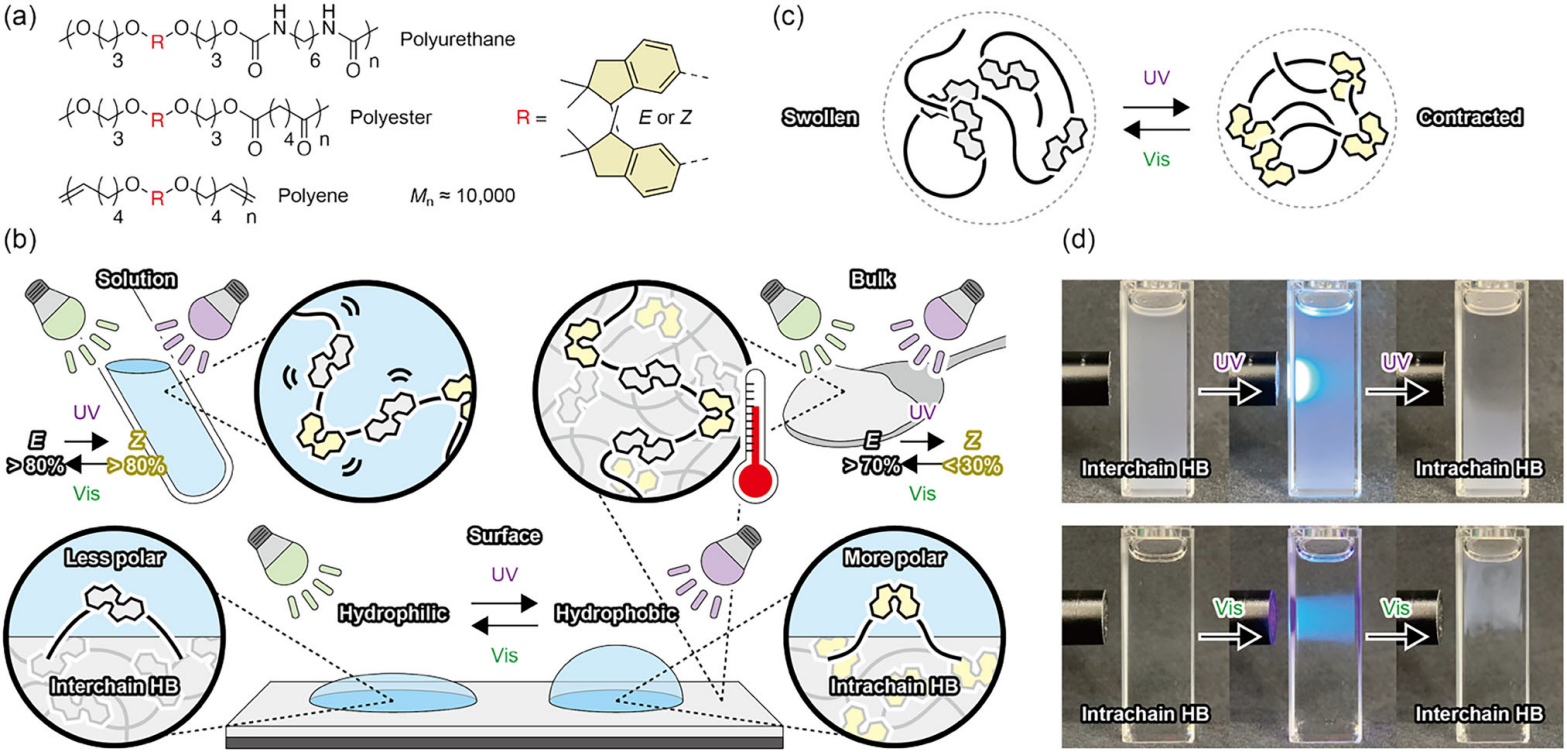
(a) Chemical structures, (b) photoresponsive behavior in solution, in bulk, and at thin‐film surfaces, (c) macromolecular conformational changes, and (d) solubility changes of linear polymers bearing *E* or *Z* isomers of HSS as repeating units in the main chains.

Next, we installed multiple HSS photoswitches as repeating units in polymer main chains and investigated their photoresponsive behavior in solution, in bulk, and at thin‐film surfaces (Figure 5) [292]. Conformations of single polymer chains have previously been controlled in solution and in bulk materials by incorporating photoswitches that undergo large structural changes, such as ABs [308], *α*‐bisimines [309, 310], and hydrazones [306], into the polymer backbones. However, the photoresponsive behavior of such systems remains insufficiently explored: the thermal instability of AB and *α*‐bisimine photoswitches likely hampers detailed studies, and the only report employing hydrazone photoswitches has been limited to studies in solution. We synthesized three types of linear polymers—polyurethane, polyester, and polyene—by step‐growth polymerizations, namely polyaddition, polycondensation, and acyclic diene metathesis (ADMET) polymerization, respectively (Figure 5a). In these polymers, different chemical linkages bridged repeating units containing either the *E* or *Z* isomer of HSS at the C6 and C6′ positions, thereby maximizing conformational changes of the single polymer chains. No side reactions, including *E*–*Z* isomerization, were observed during ADMET polymerization using a Grubbs catalyst, owing to the steric hindrance of HSS. The *M*_n_s of the obtained polymers were approximately 10,000. In solution, all polymers exhibited photoisomerization yields exceeding 80% in both *E*–*Z* directions, together with good photofatigue resistance, upon irradiation with 300 or 340 nm UV light and 405 nm visible light (Figure 5b). Moreover, the polymer conformations, as reflected by their hydrodynamic volume, were reversibly transformed between swollen (*E*) and contracted (*Z*) states, precisely depending on the *E*/*Z* ratio (Figure 5c). In the case of the polyurethanes, these conformational transformations were accompanied by switching between interchain (*E*) and intrachain (*Z*) hydrogen bonding (HB), and these nanoscopic changes were amplified into macroscopic changes in solubility (transmittance), resulting in reversible transitions between dissolved (transparent) and precipitated (opaque) states (Figure 5d). To the best of our knowledge, this represents the first example of photoswitching of main‐chain HB. The nanoscopic changes were also translated into macroscopic changes in the surface wettability of their spin‐coated polymer thin films (Figure 5b). Notably, the direction of the wettability change was opposite to that expected from the polarity difference between the *E* and *Z* isomers: the less polar *E* isomer led to a hydrophilic surface, whereas the more polar *Z* isomer resulted in a hydrophobic surface. This counterintuitive behavior also originates from the distinct HB modes, namely interchain HB in the *E* state and intrachain HB in the *Z* state. In the glassy bulk state of all polymers, the *Z*‐to‐*E* photoisomerization yields exceeded 70%, comparable to those observed in solution and extraordinarily high despite the restricted molecular mobility (Figure 5b). By contrast, the *E*‐to‐*Z* photoisomerization yields were below 30%. Although the origin of this asymmetric behavior remains unclear, the thermally bistable HSSs enabled the first quantitative studies on two‐way photoisomerization of main‐chain photoswitches in the bulk state and uncovered this previously unrecognized phenomenon.

## 4 Summary and Outlook

The present account has summarized recent progress in thermally stable photoswitches—particularly SS and HSS—and illustrated how these systems enable diverse polymer applications, largely based on our own contributions. Looking ahead, thermally stable photoswitches such as SS and HSS offer promising opportunities to further advance polymer science by enabling precise spatiotemporal control over polymer structures and properties across different states of matter. Continued molecular design to enhance structural changes, thermal stability, photoisomerization yields, and photofatigue resistance of photoswitches, together with control over their interactions with polymer chains, will broaden their applicability and deepen our understanding of photoresponsive polymeric systems. Currently, we are focusing on developing new SS‐based photoswitches and on elucidating photoresponsive behavior of HSS small molecules bearing various substituents and HSS‐containing polymers with diverse architectures. We are also interested in reversible photoinduced transitions between crystalline solid or glassy states and the liquid state and in their applications, including photoswitchable adhesives and light‐driven actuators [161, 184, 311], which have so far been achieved only in AB [312, 313], DAE [314], and hydrazone [63, 65] small molecules, as well as in AB‐based polymers [315, 316, 317]. I believe that this account will provide useful perspectives for the rational design of thermally stable photoswitches and inspire future efforts toward the development of next‐generation photoswitches and advanced photoresponsive polymeric materials with programmable functions.

## Funding

This work was supported by Japan Science and Technology Agency (JPMJPR21N2), Japan Society for the Promotion of Science (JP19K15623, JP21H01987, JP21H05884, and JP25K01834), Ministry of Education, Culture, Sports, Science and Technology (A6501) and HIRAKU‐Global Program, Strategic Professional Development Program for Young Researchers, MEXT.

## Conflicts of Interest

The author declares no conflicts of interest.
